# Left Ventricular Unloading in Extracorporeal Membrane Oxygenation: A Clinical Perspective Derived from Basic Cardiovascular Physiology

**DOI:** 10.1007/s11886-024-02067-w

**Published:** 2024-05-07

**Authors:** I. Protti, M. P. J. van Steenwijk, P. Meani, L. Fresiello, C. L. Meuwese, D. W. Donker

**Affiliations:** 1 Departments of Cardiology and Intensive Care, Erasmus University Medical Center, Rotterdam, the Netherlands; 2 Department of Pathophysiology and Transplantation, University of Milan, Milan, Italy; 3 Maastricht University Medical Center+, Cardiothoracic Surgery, Heart and Vascular Center, Maastricht, the Netherlands; 4 Cardiovascular and Respiratory Physiology, TechMed Center, University of Twente, Hallenweg 5, 7522 NH Enschede, The Netherlands; 5 Intensive Care Center, University Medical Center Utrecht, Utrecht, the Netherlands

**Keywords:** Veno-arterial extracorporeal membrane oxygenation (V-A ECMO), Extracorporeal life support (ECLS), Left ventricular unloading, Mechanical circulatory support (MCS), Temporary mechanical circulatory support (TCS), Computational physiological modeling, Impella, Intra-aortic balloon pump (IABP)

## Abstract

**Purpose of Review:**

To present an abridged overview of the literature and pathophysiological background of adjunct interventional left ventricular unloading strategies during veno-arterial extracorporeal membrane oxygenation (V-A ECMO). From a clinical perspective, the mechanistic complexity of such combined mechanical circulatory support often requires in-depth physiological reasoning at the bedside, which remains a cornerstone of daily practice for optimal patient-specific V-A ECMO care.

**Recent Findings:**

Recent conventional clinical trials have not convincingly shown the superiority of V-A ECMO in acute myocardial infarction complicated by cardiogenic shock as compared with medical therapy alone. Though, it has repeatedly been reported that the addition of interventional left ventricular unloading to V-A ECMO may improve clinical outcome. Novel approaches such as registry-based adaptive platform trials and computational physiological modeling are now introduced to inform clinicians by aiming to better account for patient-specific variation and complexity inherent to V-A ECMO and have raised a widespread interest.

**Summary:**

To provide modern high-quality V-A ECMO care, it remains essential to understand the patient's pathophysiology and the intricate interaction of an individual patient with extracorporeal circulatory support devices. Innovative clinical trial design and computational modeling approaches carry great potential towards advanced clinical decision support in ECMO and related critical care.

## Introduction

Cardiogenic shock (CS) is a complex and heterogeneous disorder characterized by a low-cardiac output state, resulting in a condition of life-threatening end-organ hypoperfusion and often culminating in multi-organ failure [1].

Veno-arterial extracorporeal membrane oxygenation (V-A ECMO) is commonly used as life-saving support for patients with refractory CS, especially in a peripheral configuration [2]. Despite its beneficial effects on organ perfusion [3], the infusion of extracorporeal blood into the aorta can also considerably increase afterload, leading to pulmonary edema, as well as intracavitary and aortic root thrombosis, all hampering the heart’s ability to recover [4]. Indeed, the absence of a therapeutic effect of V-A ECMO in a recent trial on patients with acute myocardial infarction induced CS [5] may at least in part be well explained by the failure to properly apply left ventricular unloading techniques.

Several mechanical devices, placed as adjunct circulatory support to V-A ECMO, can unload the left ventricle [6••]. Their unloading potential varies depending on the device used and the patient category [7]. Nevertheless, observational studies have suggested a survival advantage of mechanical left ventricular unloading used in addition to V-A ECMO [8].

In this manuscript, we will review different mechanical unloading techniques adjunctive to V-A ECMO through the perspective of Pressure-Volume (PV) loops. For this reflection, we will first summarize some basic concepts of PV loops in physiological conditions and in cardiogenic shock supported by V-A ECMO.

## Cardiac Physiology and Pressure-Volume Loops

**Fig. 1:**
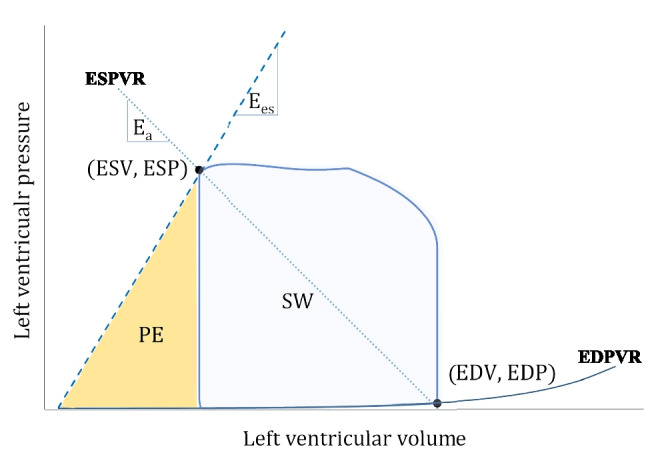
Pressure – volume loop for the left ventricle including arterial elastance (E_a_), end-systolic ventricular elastance (E_es_), Stroke Work (SW), Potential Energy (PE), end-systolic volume and pressure (ESV, ESP), end-diastolic volume and pressure (EDV, EDP), end-systolic pressure-volume relationship (ESPVR) and end-diastolic pressure-volume relationship (EDPVR). PE indicates potential energy, SW indicates stroke work. The sum of PE and SW equals the pressure volume area (PVA), see text for details

Ventricular pressure-volume (PV) loops describe temporal changes in chamber volume (x-axis) and pressure (y-axis) that occur throughout the course of each cardiac cycle. A normal cardiac cycle in a PV loop is typically demarcated by four boundaries: 1) ventricular filling on the lower part; 2) isovolumetric contraction on the right side; 3) ventricular ejection on the top; 4) isovolumetric relaxation on the left [9], as indicated in Fig. 1. The shape and position of each individual loop in the PV diagram is largely determined by the intrinsic properties of the myocardium (inotropism and lusitropism) and extrinsic hemodynamic conditions (preload and afterload).

Ventricular preload, reflecting the maximal sarcomere stretch just before the isovolumetric contraction [10], is best indexed by the End-Diastolic Volume (EDV) in a PV loop [11]. Ventricular afterload refers to the maximal myocardial wall tension during systole and translates into the pressure that the ventricle must overcome to eject blood into the aorta or pulmonary artery. In the absence of a (sub)valvular stenosis, this pressure depends on the total systemic or pulmonary vasculature load, exerted on the ventricular myocardium [12]. The latter can be approximated by the concept of effective arterial elastance (E_a_) [13, 14], the negative slope intersecting ventricular elastance (E_es_) at the End-Systolic Pressure (ESP) point (upper left corner of the loop) and the volume-axis at EDV [15] (Fig. 1). Changes in preload and afterload cause a diagonal shift of the PV loop to the left or right, upwards or downwards.

Each cardiac cycle is delimited within the boundaries of two lines, reflecting the ventricular intrinsic properties. The first is a linear association that connects all the ESP points belonging to multiple PV loops, experimentally obtained by changing preload and afterload at constant myocardial contractility [16]. The slope of this End-Systolic Pressure-Volume Relationship (ESPVR) describes the end-systolic ventricular elastance (E_es_) and approximates myocardial contractility [17]. The lower boundary of each cardiac cycle falls on a curvilinear relationship enclosing all the End-Diastolic Pressure (EDP) and EDV points of multiple PV loops obtained across different preloads. This End-Diastolic Pressure-Volume Relationship (EDPVR) describes the passive relaxation properties of the myocardium [18]. The non-linear shape illustrates the fact that ventricular compliance decreases at higher chamber volumes [19].

The ESPVR and EDPVR are used to illustrate not only the functional characteristics of the heart, but also to visualize other relevant concepts in this context. For instance, the area enclosed between the ESPVR, EDPVR and the isovolumetric relaxation line together with the area of the PV loop is known as the Pressure-Volume Area (PVA), which approximates the total mechanical energy produced by the ventricle during the entire cardiac cycle(Fig. 1) [20]. The PVA also has a highly linear correlation with myocardial oxygen consumption (MVO_2_) [21]. Of the total mechanical energy expenditure, only a small proportion is effectively consumed for blood ejection. This external mechanical work corresponds to the area within the PV loop itself and is referred to as Stroke Work (SW) [21], which normally accounts for about 25% of the total energy [22]. The remaining area (PVA minus SW) indicates the Potential Energy (PE) stored in the elastic myofilaments at the end of systole is considered to be dissipated as heat [23, 24]. A graphical representation of these areas is visualized in Fig. 1. Under physiological and resting conditions, optimal mechanical efficiency, as expressed by the SW/PVA ratio, is achieved when ventricular and arterial elastances are matched with an E_a_/E_es_ ratio close to 0.5–0.7 [25].

## Cardiogenic Shock and V-A ECMO from a PV Loop Perspective

**Fig. 2:**
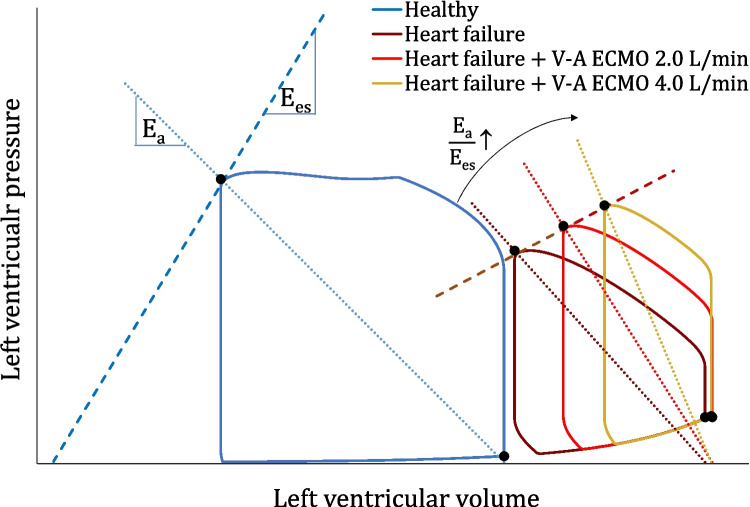
Pressure – volume loops of the left ventricle in healthy condition, during systolic heart failure and supported by V-A ECMO support at 2.0 L/min and 4.0 L/min of extracorporeal blood flow. E_a_ indicates arterial elastance (E_a_) and E_es_ left ventricular end-systolic elastance

The analysis of PV loops in CS patients provides a complementary tool for clinicians to fully understand the hemodynamic derangements underlying an individual's shock state and, therefore, allows one to tailor an appropriate therapy. In cases of CS due to profound and irreversible left ventricular (LV) dysfunction, the ESPVR flattens, mirroring a depression in myocardial contractility. This leads to a marked reduction in SV (the loop width) and blood pressure, as indexed by a decrease in ESP and loop height. As a compensatory response, catecholamine – induced venous vasoconstriction functionally shifts blood from the unstressed to the stressed compartment, thus raising the central and pulmonary venous pressures [26] and leading to an increase in arterial blood pressure. This leads to a further rightward shift of the PV loop towards a higher EDP and EDV [27, 28]. As cardiogenic shock progresses, a systemic inflammatory response develops, ultimately leading to a pronounced reduction of total peripheral vascular resistances (TPR). As a result, all compensatory mechanisms begin to fail and a profound state of hypotension and tissue hypoperfusion sets in. Therefore, as a net effect, CS results in a narrowed, shortened, and rightward – shifted PV loop, with a flattened ESPVR [27, 28], as shown in Fig. 2.

When V-A ECMO is initiated for CS, LV afterload can rise considerably due to the (retrograde) infusion of arterialized, extracorporeal blood into the descending aorta [29]. This increase in afterload steepens the slope of the arterial elastance line. If TPR and contractility remain unchanged, to overcome the higher afterload and eject blood into the aorta, the left ventricle must rely on the Frank-Starling mechanism by increasing its preload. As a result, the subsequent LV dilatation leads to a further increase in EDP and pulmonary capillary wedge pressure, causing cardiogenic pulmonary edema [3, 11, 30]. As a net effect, LV PV loops are shifted further upward and rightward along the EDPVR, becoming progressively more narrow (decreased native SV) and taller (increased afterload pressure) [31•], as visualized in Fig. 2. In this scenario, the impaired blood oxygen saturation coming from the lungs and the increased MVO_2_ (related to a larger LV PVA despite SV reduction) may further aggravate LV dysfunction and hinder cardiac recovery. In extreme situations of LV failure and overload, the aortic valve might remain closed due to a lack of left ventricular ejection, causing blood stasis and ultimately aortic root and cavity thrombosis [3, 11, 30].

## The Pathophysiological Rationale of LV Unloading

The pathophysiological rationale of considering adjunct LV unloading relevant during V-A ECMO stems from the notion that LV overload may arise by addition of oxygenated extracorporeal blood to the patient's arterial vasculature. Notably, the retrograde flow direction of arterialized blood from a femoral or iliac artery into the descending aorta and towards the failing heart is often referred to as an important aspect of the V-A ECMO-mediated LV overload. Yet, the significance of the kinetic energy related to the unphysiologically directed extracorporeal flow remains ill-defined thus far. In this context, it should be noted that central V-A ECMO has experimentally also been shown to generate LV overload, which emphasizes the role of LV afterload for LV loading conditions mainly influenced by the addition of extracorporeal blood to the native arterial system, not necessarily due to its flow direction [32]. In this context, it should be stressed that conservative measures to reduce LV afterload and preload should always be considered as an initial step during V-A ECMO support. This notion has been reported to yield at least pathophysiologically significant benefits when aiming to unload the heart and reduce the risks of hydrostatic pulmonary edema as supported by data derived from computational physiological modeling studies [31•]. Moreover, the role of right-left ventricular interaction during V-A ECMO should not be underestimated, as a preserved right ventricular contractile function can be an important contributor to LV overload as shown in computational physiological modeling studies [33].

For what has been said so far, LV unloading is thought to contribute beneficially to ventricular remodeling processes by optimizing mechanical myocardial loading conditions and reducing myocardial oxygen expenditure, thus promoting recovery of the failing heart during V-A ECMO and improving long-term prognosis [8]. Different temporary circulatory mechanical support devices can unload the left ventricle through different mechanisms [29]. In the next section, we will review some of these devices including the perspective of PV loops, as detailed before [19, 31•].

## Intra-Aortic Balloon Pump

The intra-aortic balloon pump (IABP) has long been considered a first line mechanical unloading modality during V-A ECMO in many centers [34•]. Compared to other devices, the IABP has a favorable risk profile [34•], is relatively cheap and easy to insert [35]. The device is placed percutaneously through the common femoral artery and positioned in the descending aorta. Cardiac support occurs through synchronized cyclic balloon inflation and deflation. At the onset of diastole, the balloon inflates and thereby increases diastolic aortic pressure, leading to an augmented blood flow to the systemic circulation and notably the coronary arteries [29]. Just before systole, the balloon deflates again and thereby creates a decrease in pressure that reduces LV afterload [36]. The latter aspect seems able to mitigate ECMO-induced increases in LV afterload [7]. In a PV loop, these effects are represented by a left and downward shift of the loop as compared to V-A ECMO support alone [31•].

The physiological benefits of the IABP are illustrated in animal studies demonstrating a significant reduction in LV afterload and improved myocardial oxygen supply demand balance [37]. In addition, studies in patients reported a reduction in mean pulmonary artery pressure and LV end-diastolic dimensions following IABP application [36, 38]. Despite these physiological improvements, observational studies with IABP as an adjunct to VA ECMO have reported conflicting results [8, 34•, 39] and no randomized trials have been reported. As such, current ELSO guidelines have explicated the absence of randomized clinical trials as an unmet medical need. To address this question, the *REMAP ECMO* (Randomized Embedded Multifactorial Adaptive Platform in ECMO; NCT05913622) was recently launched. By using a registry-based trial platform design with inherent synchronization of infrastructure and usage of Bayesian trial statistics, this project aims to study multiple patient management strategies during ECMO support. As a first embedded trial, this platform will study the effects of routine application of IABP on weaning success in V-A ECMO supported patients.

While results from the *REMAP ECMO* LV unloading study and other trials are pending, IABP is applied in a highly variable way across different hospitals [8, 34•]. Some centers advocate to routinely place an IABP in every V-A ECMO supported patient in whom no contra indications are present. Other centers however prefer to only initiate IABP in case of clinical evidence of LV overload. A downside from this approach may be that the IABP would fall short in a more profound situation of LV overload, possibly delaying a more effective unloading strategy, and it should be noted that a recent consensus document recommended the application of IABP in mild forms of LV overload [40].

## Impella^®^

From a mechanistic perspective, the Impella^®^ (Abiomed, Danvers, MA, USA) device, a trans-aortic microaxial blood pump, is a very potent adjunct LV unloading device when applied during V-A ECMO support [31•]. Depending on the specific type of Impella^®^, around 2–5 L/min of continuous blood flow can be generated by the catheter-based transaortic impeller, contributing to both systemic cardiac output and LV unloading; the latter ideally translating into mechanically and energetically more favorable conditions of the failing, potentially recovering heart [11, 31•]. The loss or reduction of isovolumetric contraction and relaxation that may occur during Impella^®^ support gives the PV loop its classical triangular shape [31•].

In the recent literature, adjunct LV unloading during V-A ECMO has been clearly advocated as based on the latest epidemiological evidence, and its timely initiation also seems advantageous [41••, 42]. The Impella^®^ device constitutes the most potent percutaneous approach for adjunct LV unloading during V-A ECMO to date [29], yet associated complication rates should not be neglected from clinical perspective of optimally individualized patient care [41••, 42]. Moreover, it should be remembered that only a minority of patients in cardiogenic shock complicating myocardial infarction, i.e., estimated 15–25%, might truly benefit from temporary mechanical circulatory support [43]. Among them, the initiation of adjunct LV unloading and the choice of a specific device should be a properly balanced clinical decision weighing potential benefits and complications. Thus far, the scientific literature does not provide clear guidance for optimally timed and tailored individualized adjunct LV unloading modalities during V-A ECMO and it is tempting to speculate whether recent trials would have shown more favorable outcome when deploying more uniform LV unloading strategies [5, 44]. To date, therefore, the use of multidisciplinary shock teams [45] and the application of existing guidelines [46] remains the best strategy for optimizing the management of LV unloading timing and modalities.

## Direct Venting

An alternative approach to unload the left ventricle is to vent blood from the pulmonary artery, left atrium or left ventricle [40]. This can be done through cannulation of one of the aforementioned compartments and anastomosing this blood flow to the ECMO circuit, or by creating an atrioseptostomy [6••, 40]. Venting of the left heart seems able to cause significant reductions in LV preload [7] and to decrease the risks for developing pulmonary edema [47], but its potency largely depends on the degree of venting. From a PV loop perspective, venting results in a left- and downward- shift of the PV loop [31•]. In addition, the width of the PV loop often significantly narrows, reflecting a reduction in stroke volume. This reduction in stroke volume carries, as a downside, an increased risk for thrombus formation, especially in patients with mechanical prostheses (in mitral position).

Two recent trials (EVOLVE-ECMO [47], and EARLY UNLOAD [48]) investigated the effects of trans-septal cannulation of the left atrium and consequent venting. EVOLVE-ECMO randomized 60 V-A ECMO supported patients with signs of left ventricular overload to early venting through a trans-septally placed cannula in the left atrium within a median 2.4 h after V-A ECMO initiation versus an approach without initial venting. Although there was no survival benefit, a significantly larger number of patients in the control arm developed pulmonary edema and nearly 80 percent of them eventually received left atrial venting [47]. EARLY UNLOAD was a single center study which was set out to find out whether venting through trans-septal cannulation would result in a 25 percent absolute mortality reduction at 30 days [48]. For this purpose, 116 patients with cardiogenic shock were randomized in a 1:1 fashion to left ventricular unloading through percutaneous trans-septal cannulation within 12 h after V-A ECMO initiation versus conventional therapy. In case of the latter, patients were allowed to receive LV unloading when developing signs of overload which eventually occurred in 50 percent of patients after a median duration of 22 h. Early unloading versus a conservative approach did not result in a different mortality rate at 30 days (46.6 vs 44.8%, respectively) nor in a higher success of weaning from V-A ECMO support or mechanical ventilation.

## Conclusion

Currently, multiple approaches are being used to unload the left ventricle during V-A ECMO support. The efficacy of these approaches can be largely inferred from pathophysiological considerations supported by computational physiological modeling approaches including comprehensive PV loop analyses. Meanwhile, randomized clinical trials on different unloading techniques are being published or ongoing. The scientific marriage of these trial data enriched with emerging pathophysiological insights will, in the coming years, teach us how to best approach the individual patient supported with V-A ECMO.
